# Candesartan Attenuates Ischemic Brain Edema and Protects the Blood–Brain Barrier Integrity from Ischemia/Reperfusion Injury in Rats

**DOI:** 10.6091/ibj.13672.2014

**Published:** 2014-10

**Authors:** Hamdollah Panahpour, Ali Akbar Nekooeian, Gholam Abbas Dehghani

**Affiliations:** 1 Dept. of Physiology and Pharmacology, Medical School, Ardabil University of Medical Sciences, Ardabil, Iran;; 2 Dept. of Pharmacology, Medical School, Shiraz University of Medical Sciences, Shiraz, Iran;; 3 Dept. of Physiology, Medical School, Shiraz University of Medical Sciences, Shiraz, Iran

**Keywords:** Blood–brain barrier, Brain edema, AT1 receptor, Candesartan

## Abstract

**Background: **Angiotensin II (Ang II) has an important role on cerebral microcirculation; however, its direct roles in terms of ischemic brain edema need to be clarified. This study evaluated the role of central Ang II by using candesartan, as an AT1 receptor blocker, in the brain edema formation and blood-brain barrier (BBB) disruption caused by ischemia/reperfusion (I/R) injuries in rat. **Methods:** Rats were exposed to 60-min middle cerebral artery (MCA) occlusion. Vehicle and non-hypotensive doses of candesartan (0.1 mg/kg) were administered one hour before ischemia. Neurological dysfunction scoring was evaluated following 24 h of reperfusion. Animals were then decapitated under deep anesthesia for the assessments of cerebral infarct size, edema formation, and BBB permeability. **Results:** The outcomes of 24 h reperfusion after 60-min MCA occlusion were severe neurological disability, massive BBB disruption (Evans blue extravasation = 12.5 ± 1.94 µg/g tissue), 4.02% edema, and cerebral infarction (317 ± 21 mm^3^). Candesartan at a dose of 0.1 mg/kg, without changing arterial blood pressure, improved neurological dysfunction scoring together with significant reductions in BBB disruption (54.9%), edema (59.2%), and cerebral infarction (54.9%). **Conclusions: **Inactivation of central AT1 receptors, if not accompanied with arterial hypotension, protected cerebral micro-vasculatures from damaging effects of acute stroke.

## INTRODUCTION

The critical role of renin–angiotensin system (RAS) in cardiovascular and fluid homeostasis is well established and some evidence exists about the role of angiotensin II (Ang II) in ischemic neuronal injury [1-3]. Ang II also involved in the pathophysiology of the stroke and large body of evidence, suggesting that ischemic stroke is associated with increased post-ischemic plasma renin secretion [2, 3]. The inhibition of angiotensin converting enzyme and blocking AT1 receptors reduced the damaging effects of the acute ischemia/reperfusion (I/R) injuries in animal stroke models [4-6]. However, neurovascular protective effects of RAS inhibition by AT1 receptor blockade against cerebral ischemic edema were not fully studied yet. 

The blood–brain barrier (BBB) is the main defendant of the central nervous system that controls its microenvironment. Cerebral ischemia activates complex sequences of pathophysiological events, and by disruption of BBB integrity, it allows the entry of unwanted and potentially toxic molecules to harm the brain [7]. BBB disruption, which happens during the acute phase of ischemia, may exacerbate brain injury by the induction of edema during reperfusion. Ischemic brain edema occurring during stroke *per se *is life threatening due to augmentation of intracranial pressure and herniation [8], and its conjunction with cerebral infarction intensifies the primary complications of ischemia injuries [9]. Developed brain edema observed after 24 hours of ischemia is mainly due to vasogenic edema caused by disruption or increased BBB permeability [10, 11]. The protection of BBB integrity reduces neuronal injury following ischemia [12]. Although the involvement of RAS was suggested in pathophysiology of ischemic events including stroke, there is limited data about the role of RAS in the alteration of BBB integrity and induction of vasogenic brain edema following focal cerebral ischemia [13]. Therefore, in the present study, the effects of AT_1_ receptor blockade on brain edema and BBB integrity following transient focal cerebral ischemia in rats were investigated.

It has been reported that AT_1_ receptor inhibition by non-hypotensive dose of losartan reduced cerebral edema and markedly prolonged survival in spontaneously hypertensive rats [13]. Pathologic remodeling of cerebral vessels that occur during chronic hypertension are said to interfere with the outcomes of neuroprotective agents [14]. To exclude these possibilities, we used the model of transient focal cerebral ischemia in normotensive rats. 

## MATERIAL AND METHODS

Male Sprague Dawley rats (270-330 g) were obtained from Central Animal House Facility of Shiraz Medical Sciences University (Shiraz, Iran). All the study protocols were approved by the Institutional Animal Ethics Committee of Shiraz Medical Sciences University, which follows the NIH Guidelines for care and use of laboratory animals (NIH declaration No. 85-23, revised in 1996). Rats had free access to water and standard rat chow *ad libitum* and were housed in a well-ventilated, temperature-controlled environment (22-24°C), humidity (40-60%), and light period (07.00-19.00).

***Induction of ischemia and reperfusion.*** All animals were anesthetized with i.p. injection of 400 mg/kg chloral hydrate. Core temperature was recorded continuously by rectal probe and maintained with a heating pad at 37 ± 1ºC. After neck surgery, the right common carotid artery was exposed through a midline neck incision. Cerebral ischemia was induced using the modified method of intraluminal middle cerebral artery (MCA) occlusion as described previously [15]. The right MCA was occluded with a 3-0 surgical nylon filament. The filament was inserted from the external carotid artery into the internal carotid artery until reaching the origin of MCA. Reperfusion of the ischemic region started after 1 h of MCA occlusion by gently removing the filament. Finally, all incisions were sutured, and the animal was allowed to recuperate during 24 h of reperfusion period. In some animals, arterial blood samples were taken from tail artery for the measurements of arterial pH and blood gases at 10 min before and 30 min after MCA occlusion as well as 10 min after the start of reperfusion. 

***Experimental protocol***. Animals were randomly divided into three main groups with 32 rats per group. Group 1 (Sham): All rats underwent the surgery at the neck region and received a single intravenous injection of the vehicle (1 ml/kg 0.1 normal sodium carbonate solution) without being exposed to brain ischemia. Group 2 (control ischemic): rats underwent the surgery at the neck region, as in the sham group, and received the vehicle 1 h before exposure to 60 min MCA occlusion followed by 24 h reperfusion. Group 3 (Candesartan-treated ischemic): all procedures performed here were similar to control ischemia with the exception of receiving a single intravenous injection of 0.1 mg/kg candesartan (Toronto Research Chemical Inc, Canada) at 1 h before induction of ischemia. 

Experiments were performed on four sets of animals (n = 8) within each group. The first set of animals was studied to determine neurological score and infarct size. The assessment of the brain edema formation was performed in the second set of animals. The third set of animals was used for the detection of BBB permeability. In the fourth set of animals, posterior tail artery was cannulated to continuously record the mean arterial blood pressure during the experiment and control of physiological parameters. 

***Evaluation of neurological outcome.*** Behavioral tests were performed in rats 24 h after the termination of MCA occlusion. A five-point grading scale of neurological deficit scoring was used as described previously [5]. In brief, rats with normal motor function were assigned as grade 1. Rats that showed flexion of contralateral torso or forelimb upon lifting by their tail received grade 2. Grade 3 was for circling to the contralateral side of the occlusion. Grade 4 was assigned to the loss of righting reflex and decreased resistance to lateral push, and finally grade 5 was for no spontaneous motor activity. 

***Cerebral infarct size. ***Cerebral infarct size was measured according to the method of Swanson *et al.* [16]. Accordingly, the brain was dissected into six 2 mm-thick slices in the coronal plane and stained with a 2% solution of 2,3,5-triphenyltetrazolium chloride (Molekula, UK) then was fixed in buffered formalin. Images of the stained sections were taken, and the infarction areas were quantified by image analyzer software (NIH Image Analyzer). Cerebral infarction volume was calculated as described previously [17].

***Blood-brain barrier*** ***permeability. ***The rats chosen for the assessment of BBB integrity were deeply anesthetized, and a catheter was placed into their lateral tail vein. Slow infusion of 2% Evans blue solution (1 ml/kg, Sigma Chemical Co., UK) was carried out for 5 min, in the sham group, 30 min after neck surgery and in the ischemic groups, 30 min after the termination of MCA occlusion. After neurological assessment, the rats were deeply anesthetized, and 250 ml warm normal saline (37ºC) was infused to the left ventricular catheter to wash out the remnants of Evans blue from general circulation. After decapitation, the brain was gently excised, the cerebellum and olfactory bulb were removed, and with the help of a brain matrix, the rest was divided into right and left hemispheres. Each hemisphere was carefully weighed and homogenized in 2.5 ml phosphate buffered saline. Evans blue absorbance was measured in the extracted supernatant at 610 nm by a spectrophotometer (UV 7500, Spectro Lab, England), and tissue Evans blue concentration (µg/g wet tissue) was calculated against a standard curve [18]. 

***Brain edema*** ***.*** The wet/dry weight method was used to measure the absolute brain water contents (ABWC). In brief, rats were killed under deep anesthesia, quickly decapitated, and the removed brain was placed in the brain matrix to separate the cerebellum and the olfactory bulb. Subsequently, through a midline sagittal incision the brain was divided into the right and the left hemispheres. Each hemisphere was placed in a separate pre-weighed container to measure its wet weight. Then, the container and the tissues were placed in a 110°C oven for 24 hours to obtain the tissues’ dry weight. ABWC of each hemisphere (%) and the percentage of edema formation (% ΔH_2_O) of the lesioned hemisphere were determined using equations 2 and 3, respectively [5].

(2) ABWC (%) = [(wet weight – dry weight)/ wet weight] × 100

(3) ΔH_2_O=%H_2_O lesioned hemisphere - %H_2_O of non-lesioned hemisphere

***Statistical analysis.*** Most of Data are presented as mean ± SEM, and the significance of differences was evaluated using one-way analysis of variance (ANOVA) followed by Tukey’s test. Neurological deficit values are given as median values with quartile range (25 -75%). Significant differences were analyzed by Mann-Whitney U Test. Statistical significance was accepted at *P*<0.05.

## RESULTS

**Fig. 1:**
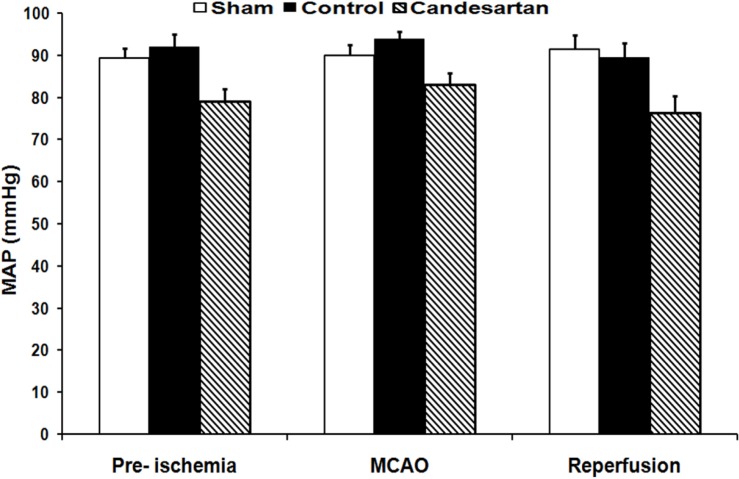
Mean arterial pressure (MAP) in sham-operated rats, vehicle-treated control, and 0.1 mg/kg candesartan-treated ischemic rats at 10 minutes before middle cerebral artery occlusion (MCAO), 30 minutes after MCAO, and 10 minutes after the beginning of reperfusion (n = 8). MCAO, Middle cerebral artery occlusion

***Mean arterial blood pressure*** *.* The results of blood pressure recordings indicated that mean arterial blood pressure of control ischemia and candesartan-treated rats were all in normal physiological range, and quantitatively no significant differences existed among them before, during, and after MCA occlusion (mean ± SEM, 92 ± 3 vs. 79 ± 2.9, 94 ± 1.6 vs. 83 ± 2.7, and 89 ± 3.5 vs. 77 ± 3.9, respectively, Fig. 1). 

***Physiological parameters.*** All physiological parameters, such as PaO_2_, SaO_2_, PaCO_2_, pH, blood glucose, and body temperature, measured during the experiment were at normal physiological range. The comparison of Inter- and intra-group indicated no significant differences among the parameters proper values.

***Neurological outcome.*** Ischemia produced severe motor disabilities in control ischemia rats. However, there was a significant improvement in neurological dysfunction scoring of ischemic rats treated with 0.1 mg/kg candesartan. The median (25-75% quartile range) of total deficit score for control ischemic group was 3 (3-3.5) compared with 1 (1.0-1.5) in candesartan-treated group (*P* = 0.002, Mann-Whitney U Test).

**Fig. 2:**
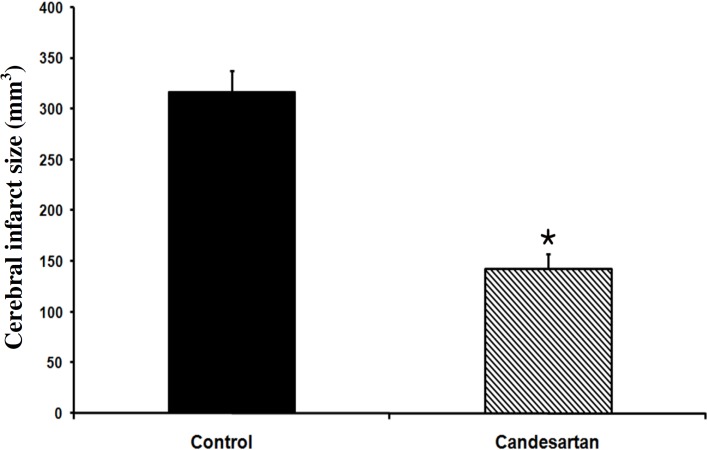
Cerebral infarct volume in vehicle-treated control and 0.1 mg/kg candesartan-treated ischemic rats (n = 8). *P<0.001, compared with control group

***Cerebral infarction. ***There were no traces of infarction in the left and right hemispheres of sham-operated rats. MCA occlusion of the right hemispheres produced cerebral lesions in control ischemia rats. However, the pre-ischemic blockade of AT1 receptor with a non-hypotensive dose of candesartan (0.1 mg/kg) significantly diminished cerebral lesion volumes by 54.9% (mean ± SEM, 143 ± 14 vs. 317 ± 21 mm^3^, *P*<0.001, Fig. 2).

**Fig. 3:**
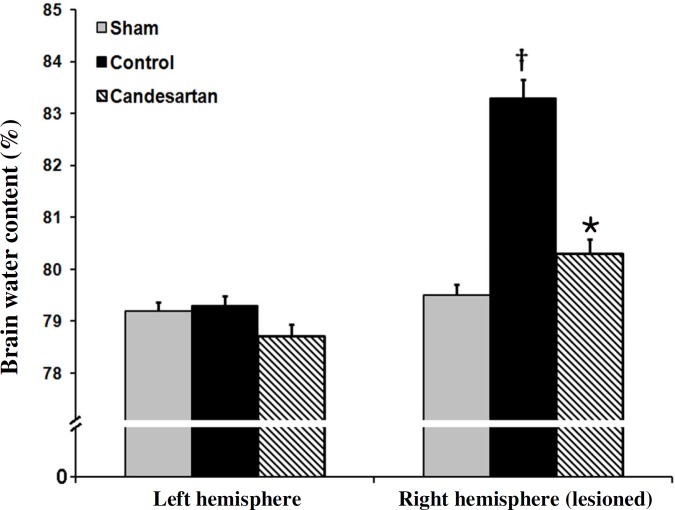
Brain water content in left side and right side of brains in sham-operated rats, vehicle-treated control, and 0.1 mg/kg candesartan-treated ischemic rats (n = 8). ^†^*P*<0.001 compared with sham group; *P<0.001 compared with control group

***Edema formation*** **. **The values of ABWC of the left and the right hemispheres of sham and left hemispheres of the ischemic rats were not statistically different (Fig. 3). Ischemia elevated brain water content of the lesioned hemispheres (right) of control ischemia rats to 83.3 ± 0.35%, while pre-ischemic blockade of AT1 receptors with candesartan (0.1 mg/kg) could significantly diminish BWC (mean ± SEM, 80.3 ± 0.28%, *P*<0.001, Fig. 3). The brain edema in ischemia control rats was significantly more than that in sham-operated rats. Pre-treatment with candesartan (0.1 mg/kg) significantly reduced the brain edema formation provoked by I/R injury by 59.2% (mean ± SEM, 1.64 ± 0.38% vs. 4.02 ± 0.32% *P*<0.001). 

**Fig. 4:**
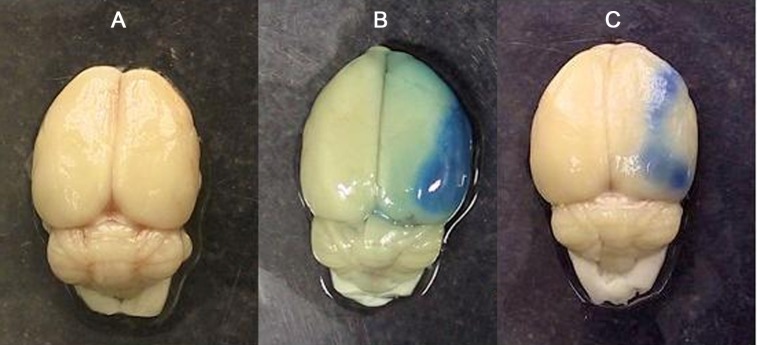
Photograph of brains in sham-operated animal (A), and ischemic rats received vehicle (B) or 0.1 mg/kg candesartan (C). Animals received Evans blue 30 minutes after surgery or middle cerebral artery occlusion (MCAO). The presence of blue color depicted in B and C indicates that extravasation of Evans blue has occurred during ischemia/reperfusion injuries in the right side of the brain and its intensity is related to the quantity of damages of the cerebral vasculature of the lesioned side

**Fig. 5:**
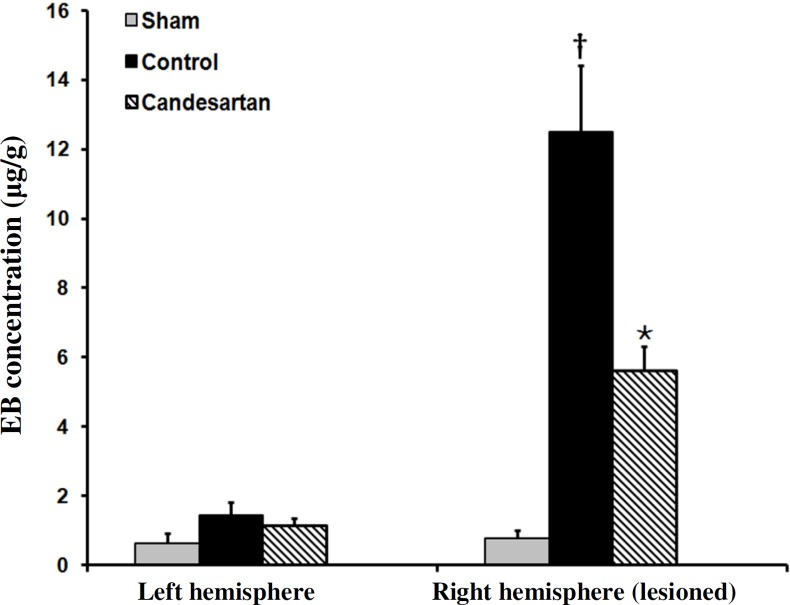
Evans blue concentration in left side and right side of the brains in sham-operated rats, vehicle-treated control, and 0.1 mg/kg candesartan-treated ischemic rats (n = 8). ^†^ *P*<0.001 compared with sham group; * *P*<0.001 compared with control group

***Blood-brain barrier*** ***integrity disruption***. Figure 4 is a sample photograph of the brain of the rats exposed to Evans blue extravasation technique. Evans blue extravasation colors the brain tissue blue in the lesioned hemispheres of ischemic rats. The absence of blue color in the right and the left hemispheres of sham (Fig. 4A) indicated that the induction of anesthesia, neck surgery, or manipulation of the carotid arteries *per se* did not affect BBB integrity. The appearances of bluish color in the lesioned hemispheres of ischemic rats indicated that the 60-min occlusion of the right MCA induced different magnitudes of BBB disruption (Fig. 4B and C). Qualitative comparisons of the blue color areas of ischemic hemispheres indicated that candesartan at dose of 0.1 mg/kg was able to attenuate BBB disruption (Fig. 4C). Figure 5 shows Evans blue concentrations of right side and left side of the brains of sham and ischemic rats receiving the vehicle or candesartan (0.1 mg/kg). There was no statistically significant difference between the Evans blue concentrations of left side brains of such groups. Moreover, there was no statistically significant difference between the Evans blue concentrations of left and right side of the brains of sham-operated rats. Evans blue concentration of ischemic hemisphere in control rats was significantly more than that in sham-operated rats. Pre-treatment with candesartan (0.1 mg/kg) significantly lowered Evans blue concentration and reduced BBB integrity disruption by 54.9% (mean ± SEM, 5.63 ± 0.7 µg/g vs. 12.5 ± 1.94 µg/g, *P*<0.01).

## DISCUSSION

The results of the present study demonstrated that induction of focal cerebral ischemia significantly increased ABWC of ischemic hemisphere. Pre-ischemic blocking of AT_1_ receptors by a non-hypotensive dose of candesartan significantly reduced ABWC in the ischemic hemisphere and lowered ischemic edema by 59.2%. There are limited reports about the direct measurement of ischemic edema formation during AT1 receptors inhibition in normotensive rats. Indirect evaluation of tissue swelling of the ischemic hemispheres using 2,3,5- triphenyltetrazolium chloride method [14, 19, 20] supports the results of the present study.

The protective effects of AT_1_ receptor blockade on ischemic brain edema have been demonstrated in other experimental models and animal species. Blezer and colleagues [13] showed that the inhibition of AT_1_ receptors by a non-hypotensive dose of losartan reduced cerebral edema and markedly prolonged survival in spontaneously hypertensive rats. Also, the inhibition of AT_1_ receptors by losartan prevented brain edema following global cerebral ischemia in the cat [21]. Moreover, the infusion of Ang II worsened cerebral edema following cerebral ischemia induced by bilateral occlusion of common carotid arteries in gerbils [22]. These results support the conclusions of the present study that Ang II and AT_1_ receptors might be involved in the formation of brain edema during I/R injuries.

Mechanisms of the beneficial effects of AT_1_ receptor blockade on ischemic brain edema have not been fully elucidated. The present study as well as that of Blezer et al. [13] showed that the protective effects of AT1 receptors blockade was achieved without hypotension. It seems that non-hypotensive mechanisms play a major role in these protective properties. Mechanisms such as the alteration of endothelial NO synthase activity and improved vasomotion of cerebral arterioles might also be involved in the protective effects of AT_1_ receptor inhibition [23, 24]. Furthermore, recent evidence suggests that Ang II may be an important stimulus for the production of superoxide and peroxynitrite in blood vessels [25]. Oxygen-derived free radicals are known to increase the permeability of the BBB [26]. Furthermore, increased Ang II via AT_1_ receptors may enhance cerebrovascular permeability and edema by the expression of matrix metalloproteinases [27]. Thus, the blocking of AT_1_ receptors may reduce ischemic edema via protective effects on BBB integrity and lowering its permeability during ischemia. Hence, the next part of this study explored the role of Ang II and AT_1_ receptors in alteration of BBB integrity during I/R injuries.

Mechanisms of the protective effects of AT_1_ receptor blockade on BBB integrity during ischemia are not fully elucidated. Several factors, such as oxygen free radicals [30], NO [31], and proteases [32], which are produced during ischemia, affect BBB permeability. The formation of free oxygen radicals in the brain tissues during or immediately after ischemia is known to increase BBB permeability [30]. Superoxide form-ation in the blood vessels by Ang II stimulation of AT_1_ receptors may also increase BBB permeability [26]. The blockade of AT_1_ receptors could, therefore, reverse the elevated BBB permeability seen during ischemia. This is possibly performed by increasing the activity of antioxidant enzymes, such as glutathione-s-transferase and superoxide dismutase [29, 33] that reduce superoxide and peroxynitrite in blood vessels [34, 25]. Therefore, the protective effects of AT_1_ receptor blockade on BBB integrity might be due to the decreased ROS production during cerebral ischemia. In conclusion, the results of this study demonstrated that the pre-ischemic blockade of AT_1_ receptors by candesartan reduced vasogenic brain edema formation by protecting the integrity of BBB following focal cerebral ischemia in the normotensive rats. 
